# High-generation near-isogenic lines combined with multi-omics to study the mechanism of polima cytoplasmic male sterility

**DOI:** 10.1186/s12870-021-02852-7

**Published:** 2021-03-05

**Authors:** Benqi Wang, Zunaira Farooq, Lei Chu, Jie Liu, Huadong Wang, Jian Guo, Jinxing Tu, Chaozhi Ma, Cheng Dai, Jin Wen, Jinxiong Shen, Tingdong Fu, Bin Yi

**Affiliations:** National Key Laboratory of Crop Genetic Improvement, National Center of Rapeseed Improvement, Huazhong Agricultural University, Wuhan, 430070 China

**Keywords:** Polima, CMS, Multi-omics analysis, *Brassica napus*, *orf224*, *Rfp*

## Abstract

**Background:**

Cytoplasmic male sterility (CMS), which naturally exists in higher plants, is a useful mechanism for analyzing nuclear and mitochondrial genome functions and identifying the role of mitochondrial genes in the plant growth and development. Polima (*pol*) CMS is the most universally valued male sterility type in oil-seed rape. Previous studies have described the *pol* CMS restorer gene *Rfp* and the sterility-inducing gene *orf224* in oil-seed rape, located in mitochondria. However, the mechanism of fertility restoration and infertility remains unknown. Moreover, it is still unknown how the fecundity restorer gene interferes with the sterility gene, provokes the sterility gene to lose its function, and leads to fertility restoration.

**Result:**

In this study, we used multi-omics joint analysis to discover candidate genes that interact with the sterility gene *orf224* and the restorer gene *Rfp* of *pol* CMS to provide theoretical support for the occurrence and restoration mechanisms of sterility. Via multi-omics analysis, we screened 24 differential genes encoding proteins related to RNA editing, respiratory electron transport chain, anther development, energy transport, tapetum development, and oxidative phosphorylation. Using a yeast two-hybrid assay, we obtained a total of seven *Rfp* interaction proteins, with *orf224* protein covering five interaction proteins.

**Conclusions:**

We propose that *Rfp* and its interacting protein cleave the transcript of *atp6/orf224*, causing the infertility gene to lose its function and restore fertility. When *Rfp* is not cleaved, *orf224* poisons the tapetum cells and anther development-related proteins, resulting in *pol* CMS mitochondrial dysfunction and male infertility. The data from the joint analysis of multiple omics provided information on pol CMS’s potential molecular mechanism and will help breed *B. napus* hybrids.

**Supplementary Information:**

The online version contains supplementary material available at 10.1186/s12870-021-02852-7.

## Background

Cytoplasmic male sterility (CMS) refers to plants’ inability to yield functional pollen, and this natural occurrence is widespread in vascular plants, specifically in flowering plants [1]. Researchers use CMS to adequately breed crops for hybrid seed production, which is a highly effective approach to use heterosis [2]. CMS appears to be mainly caused by rearrangements, deletions, or mutations of genes located in mitochondria, which can be eliminated by the fertility restoration factor genes present in the nuclear genome [3, 4]. CMS not only can be used to study the relationship between cytoplasmic genes and nuclear genes but can also be a suitable donor for examining heterosis, providing a theoretical basis for three-line breeding [5, 6]. As researchers continue to elucidate the factors that lead to abnormal pollen development, several factors can cause this phenomenon to be discovered. Scientists have made corresponding assumptions regarding CMS mechanisms, such as cytotoxicity, advanced programmed cell death (PCD), energy deficit, and reverse inverse regulation hypotheses [7]. Although there is no direct evidence to prove the toxic protein hypothesis, the fertility suppression effect of sterility genes on prokaryotic cells has been well documented [8–10]. Rearrangement of mitochondrial genes leads to energy loss, which might be a significant reason for anther abortion in CMS [11]. Previous studies have shown that during the CMS-T of maize, the mitochondria split rapidly during anther development. Therefore, elevated expression of CMS genes might cause mitochondrial function defects, resulting in insufficient energy supply for male organ development, thus triggering abortion [12]. ATP synthesis requires the flow of hydrogen ions generated by the concentration gradient of hydrogen ions produced by complex I, II, III, and IV in the mitochondria, and the integrity of the mitochondrial membrane structure is crucial to the generation of ATP [[11, 13]]. Many proteins encoded by CMS genes, including *URF13, ORF138, ORF79*, and *ORPH79,* are transmembrane proteins. These proteins may bind to the mitochondrial inner membrane, affecting the hydrogen ion concentration gradient, thus affecting ATP synthesis [14–16].

Oil-seed rape (*Brassica napus L.*) is the fourth-largest oil-producing crop worldwide. The cytoplasmic male sterility of *Brassica napus* has also been extensively studied as a rich source of cytoplasmic male sterility promoting hybrid seed production for the desirable traits. Previous studies found that there are at least nine types of CMS in rapeseed: ogu CMS [17], nap CMS [18], Tour CMS [19], *pol* CMS [20], *shan2A* CMS [21], *oxa* CMS [22], *Nca* CMS [23], *Moricandia arvensis* CMS [20], *Nsa* CMS [24], *hau* CMS [25], *inap* CMS [25]. CMS is classified into sporophytic and gametophytic sterility according to the period of anther abortion. In rapeseed, the currently reported type of sterile cytoplasm is sporophytic sterility. According to the cytoplasm source, CMS is branched into two types: homogeneous cytoplasm and heterogeneous cytoplasmic sterility [26].

In rapeseed, the most familiar type of CMS is *pol* CMS, which is used worldwide for research purposes. Since its discovery in 1987, it has been found that abnormal transcription of the mitochondrial gene *orf224/atp6* leads to the occurrence of *pol* CMS [27], but how the sterility gene *orf224* contributes to the development of abnormal anther functioning remains to be explored [28]. Besides, the restoration gene of *pol* CMS has been verified by map-based cloning [29–31]*. Rfp* (fertility-restorer) is a nuclear gene encoding a PPR protein located in mitochondria, and it ultimately leads to the malfunctioning of the sterility gene *orf224*, thus allowing restoration of pol CMS-induced fecundity [29]. However, the mechanism of fertility restorer is not scrutinized. Anther examination in *B. napus* showed that some proteins’ cumulative levels fluctuate considerably, including proteins related to energy and carbohydrate metabolisms, photosynthesis and flavonoid production, aldehyde dehydrogenase performance, and cell wall remodeling [32]. CMS’s occurrence is characterized by considerable differences in protein level, transcription level, and metabolic level. We will further analyze CMS’s causes and results through the combined analysis of proteomes, transcriptomes, and metabolomes to provide a new research basis for elucidating nuclear-plasmid interfaces. The discovery of new types of CMS may not only improve the cytoplasmic resources of cruciferous plants but also provide valuable resources for examining nuclear-plasmid interaction [11, 33]. In the development of multi-omics, nucleomics (including genomics and transcriptomics) reveal the underlying factors of life phenomena, proteomics reveals the external factors of life phenomena, and metabolomics reveals the final results phenomena. Therefore, by integrating the results of different monolayers of histology to realize the deeper excavation of the essence of life phenomena from cause to effect and from the surface to inward, we will have a more comprehensive and accurate understanding of the laws that reveal the characteristics of life [[34, 35]].

RNA is a genetic macromolecule that plays numerous roles in eukaryotes and prokaryotes. RNA has three types, which have different locations and functions within the cell during an organism’s lifecycle Messenger RNA (mRNA), Ribosomal RNA (rRNA), and transfer RNA (tRNA). RNA is a significant element of ribozymes and riboproteins. The microdissection technique for isolating specific materials is required for gene expression profiling and has become a popular research area. Unlike single-celled organisms, higher plants have evolved into too complex organisms; they are categorized based on the essential functions performed by well-maintained organs and tissues. Current researchers ignore this point that plants have different cell types; therefore, their experimental results have not been in-depth or informative. Analysis of overall transcription levels with a high spatial resolution is mandatory to describe specific tissues’ status entirely. Therefore, differences in gene transcription at the single-cell level that occur in specialized plant cells are crucial for analyzing the mechanism of plant trait occurrence, and these differences have also sparked widespread interest among scientists [36]. Laser capture microdissection, laser microdissection, and pressure ejection are suitable sampling techniques for collecting homogeneous cells to analyze plant metabolites. Tapetum cells were excised by microdissection, and RNA was extracted and amplified to perform single-cell transcriptome sequencing. In the present study, we applied a multi-omics joint analysis to discover candidate genes that interact with the sterility gene *orf224* and the restorer gene *Rfp* of *pol* CMS to provide theoretical support for the occurrence and restoration mechanisms of sterility.

## Results

### Observation of micromorphological defects of pollen CMS in *Brassica napus*

**Fig. 1:**
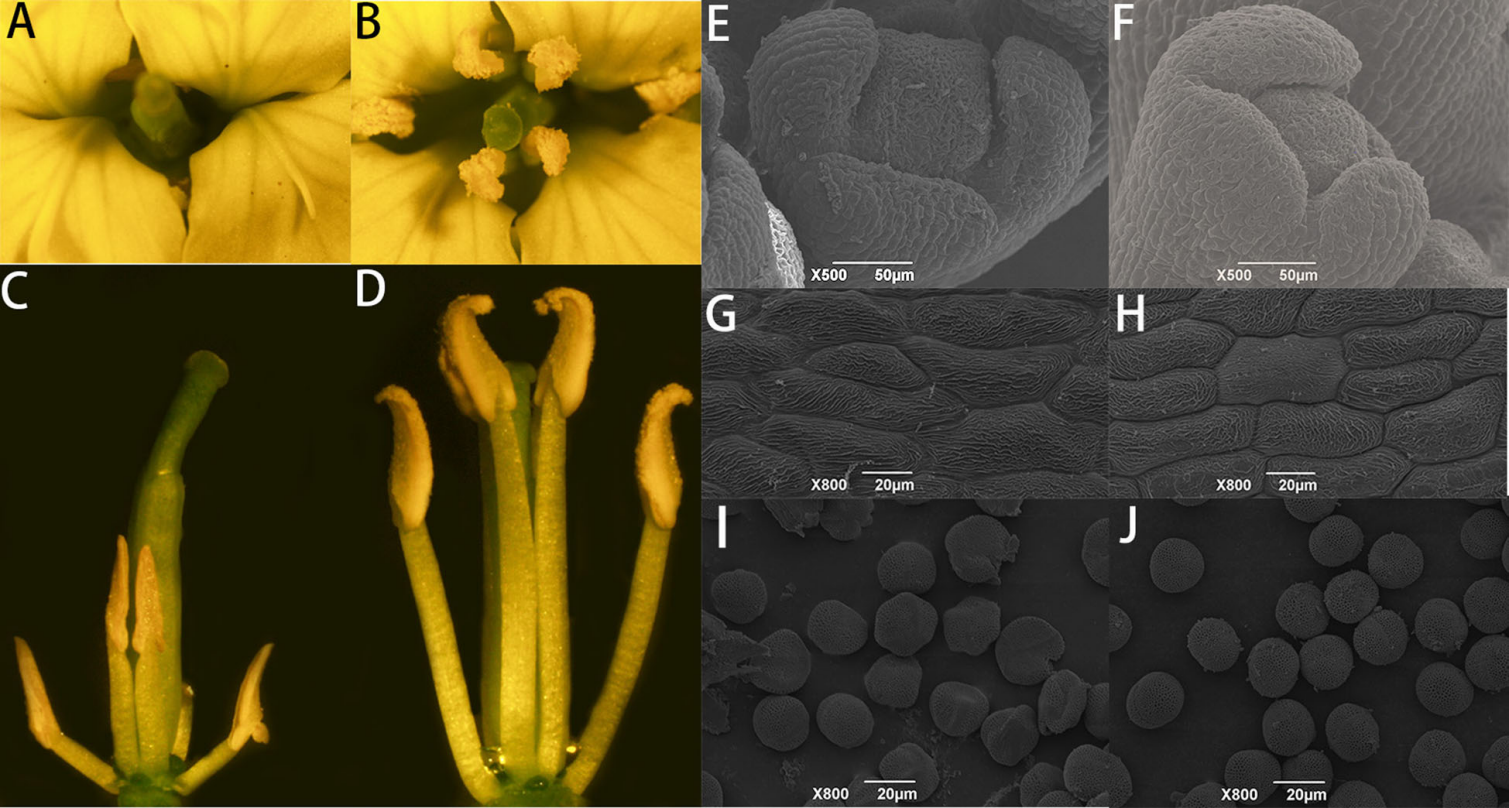
Stereomicroscopy and scanning electron microscopy analyses of differences in anther development and pollen development in sterile and reintroduction materials. (A, C, E, G, I) *pol* CMS sterile plants anthers, flower buds, and pollen grains in the corresponding period. (B, D, F, H, J) *pol* CMS recultivated plant stamens mature anthers, flower buds, pollen grains during differentiation

We used the sterile line 1141A of *pol* CMS and the restoring line material bing409. After 13 generations of backcrossing, we constructed the NIL material of the restoration gene of *pol* CMS. There was no phenotypic difference between the sterile line and the restorer line throughout the asexual development period. However, in the later period of the restorer growth, the *pol* CMS’s flower buds and flowers’ morphology differed significantly. After stereomicroscopy and scanning electron microscopy observation of the difference between the anther and pollen of the sterile line and restorer line, we found a considerable difference in pollen between the sterile and restorer lines. In the sterile line, the buds and stamens were dried up, the petals became smaller and contracted, and there was no pollen and significantly less nectary (Fig. 1 A and C).

In contrast, the restored plant was healthy, showing a wild-type petal and stamen growth with a maximum amount of nectary (Fig. 1 B and D). Comparison of sterile-line pollen with restorer-line pollen via scanning electron microscopy revealed many abnormal developmental protrusions (Fig. 1 E and F), anther epidermal growth retardation (Fig. 1 G and H) with pollen grains present on the surface, and abnormal shrinkage rupture (Fig. 1 I and J). The pollen grains of sterile plants shrank markedly, bent inward, and collapsed; moreover, the outer wall development was abandoned, and the central plate was irregular.

### Analysis of anther metabolites in sterile and restorer lines of *pol* CMS near-isogenic lines

**Fig. 2:**
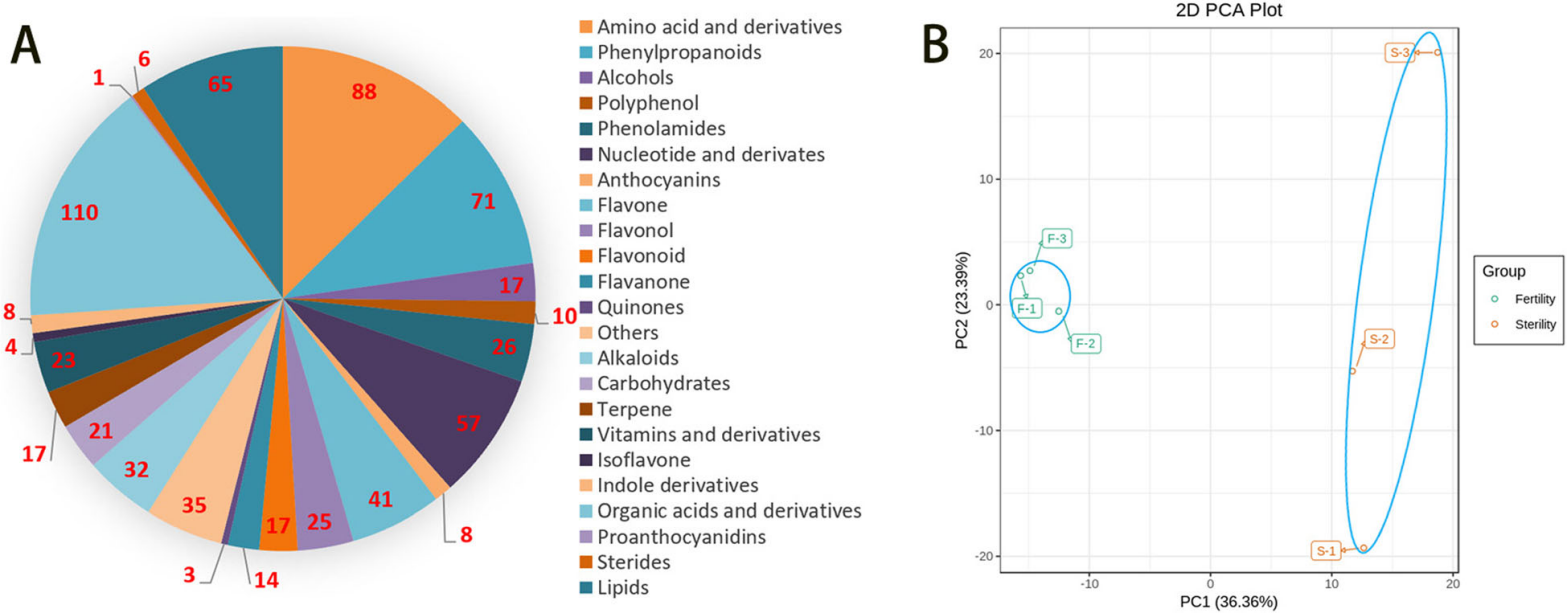
Overview of metabolomics data. **a** The metabolome type and the number of species in each group. **b** The principal component analysis showed significant differences between the two traits

Metabolic analysis of the flower buds of sterile and restorer lines of *pol* CMS near-isogenic lines identified 699 metabolites, including 88 amino acids derivatives, 71 phenylpropanoids, 17 alcohols, 10 polyphenols, 26 phenol amides, 57 nucleotide derivatives, 8 anthocyanins, 41 flavones, 25 flavonols, 17 flavonoids, 14 flavanones, three quinones, 32 alkaloids, 21 carbohydrates, 17 terpenes, 3 vitamins and derivatives, four isoflavone, 8 indole derivatives, 110 organic acids and derivatives, one proanthocyanidin, six steroids, 65 lipids, and 35 others (Fig. 2a). Principal component analysis (PCA) showed that the abundance of the metabolites in the flower buds of sterile and restorer lines of *pol* CMS changed tremendously, and the sterile and fertile were well separated, with the largest reaching 23.39% and the groups reaching 36.36% (Fig. 2b).

**Fig. 3:**
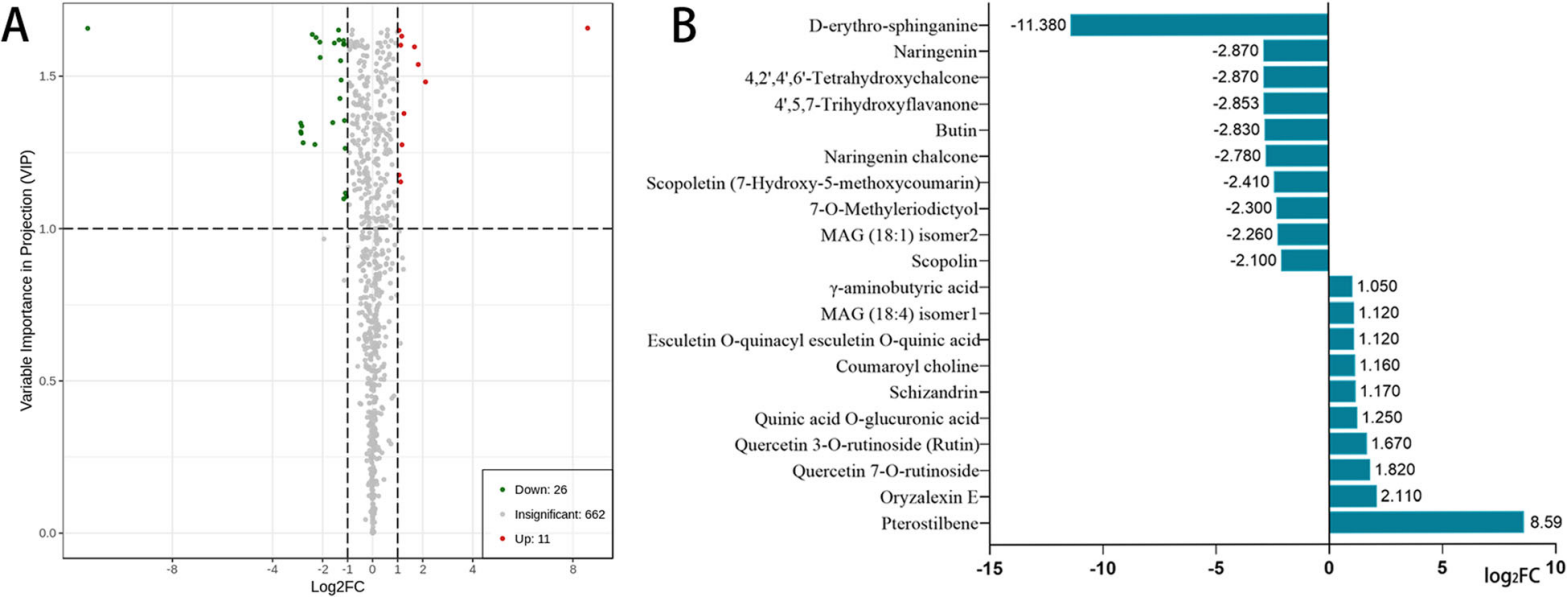
Differences in metabolites between sterile and restorer lines in the metabolome. **a** Volcano plot of differential metabolites of the metabolome. **b** Histogram of the top 20 substances in the differential metabolites of the metabolome

In this study, by using heat map clustering, we classified the metabolites of the sterile and restorer lines (Fig. S1A). KEGG enrichment analysis showed that the metabolites were involved in secondary metabolism, flavonoid metabolism, and glycolysis/gluconeogenesis pathways, as the metabolite levels changed remarkably in these pathways (Fig. S1B; Table S1). A volcano plot of differential metabolites showed 37 metabolite differences (|Fold change| ≥ one and |Fold change| ≤ 0.5) between the sterile and restorer lines and 11 kinds of flower buds in the sterile line compared with those in the restorer line. Moreover, there were 11 up-accumulated and 26 down-accumulated metabolites (Fig. 3a). Figure 3b lists the top 20 substances with multiple metabolite differences, with pterostilbene being the most up-accumulated and D-erythro-sphinganine being the most down-accumulated (Table S4).

### Differentially abundant proteins (DAPs) between the sterile and restorer lines of *pol* CMS

To further explore the mechanism of *pol* CMS in the sterile and restorer lines, we sampled the flower buds of *B. napus* < 0.5 mm to determine the proteomes, and we used a label-free method to detect the gene expression differences and protein abundance differences between sterile and restorer lines of near-isogenic lines. For proteomes, 711,479 total spectra, 27,274 matched spectra, 46,998 peptides, 30,391 unique peptides, 7598 proteins, and 4967 quantifiable proteins were detected (Fig. S2A,C). PCA analysis results showed that among the sterile and restorer lines, the difference between the groups was significant at 44.6%, and the difference within the group was 14.9%, which can better distinguish the differences between the two materials and indicates that the method has good quantitative repeatability (Fig. S2B).

**Fig. 4:**
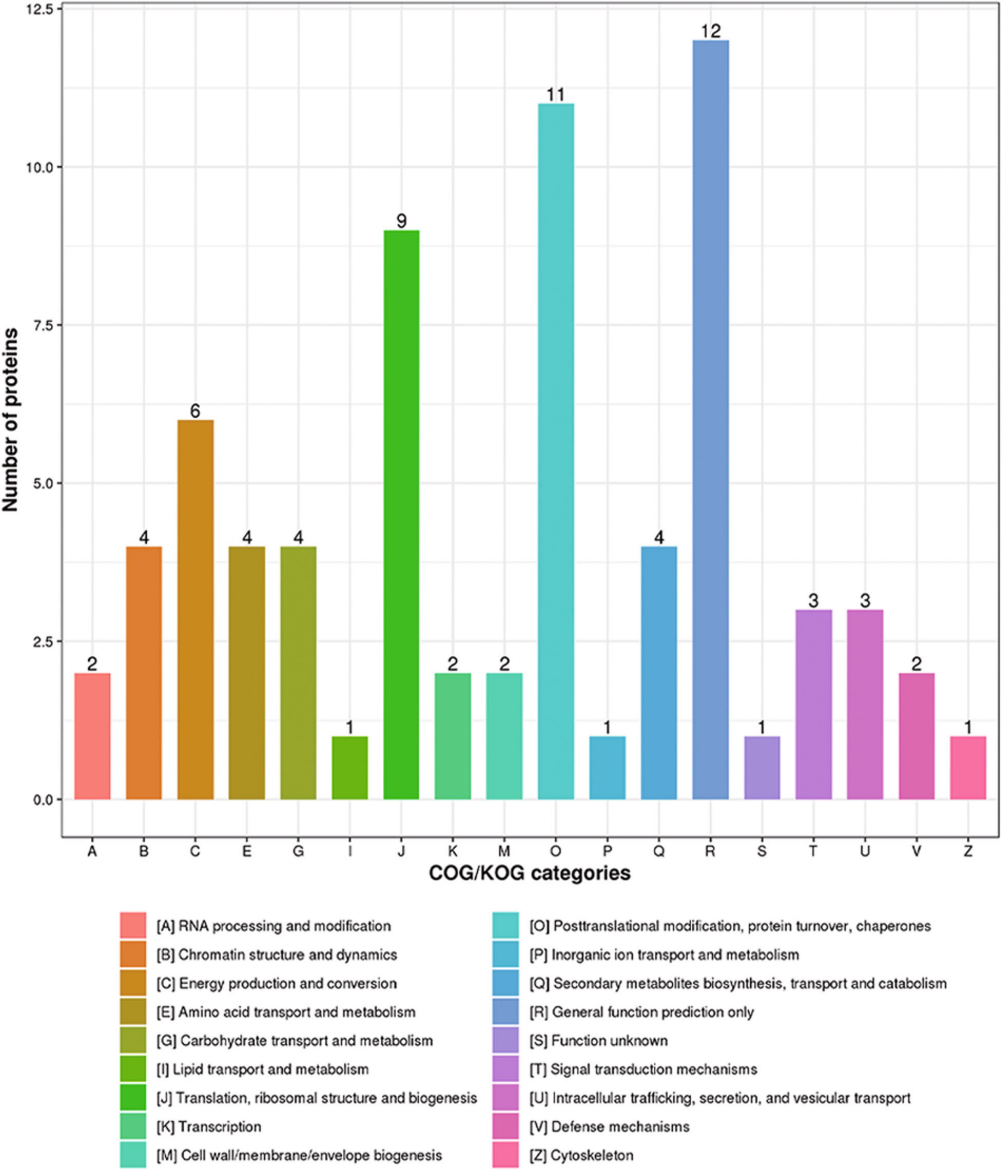
Differentially abundant proteins (DAPs) between the sterile line and its iso-nuclear maintainer line. Volcano plot of DAPs (fold > 1.5)

The label-free method was used to detect proteins at four levels. Based on -log10 *P*-value > 1.5, 140 DAPs were identified. Among these proteins, 65 were up-accumulated, and 75 were down-accumulated (Fig. S2D). COG/KOG classification analysis showed that DAPs were divided into 18 functional (Fig. 4). COG/KOG functional analysis of differentially expressed proteins showed that metabolic processes with considerable differences involved post-translational modification, protein turnover, chaperones, translation, ribosomal structure and biogenesis, and energy production and conversion (Table S3, S5). Through previous studies, it is found that *pol* CMS’s occurrence mainly occurs at the beginning of the fourth stage of anther development. The tapetum layer is caused by programmed death in advance, and the tapetum layer mainly provides energy for the development of anthers.

**Fig. 5:**
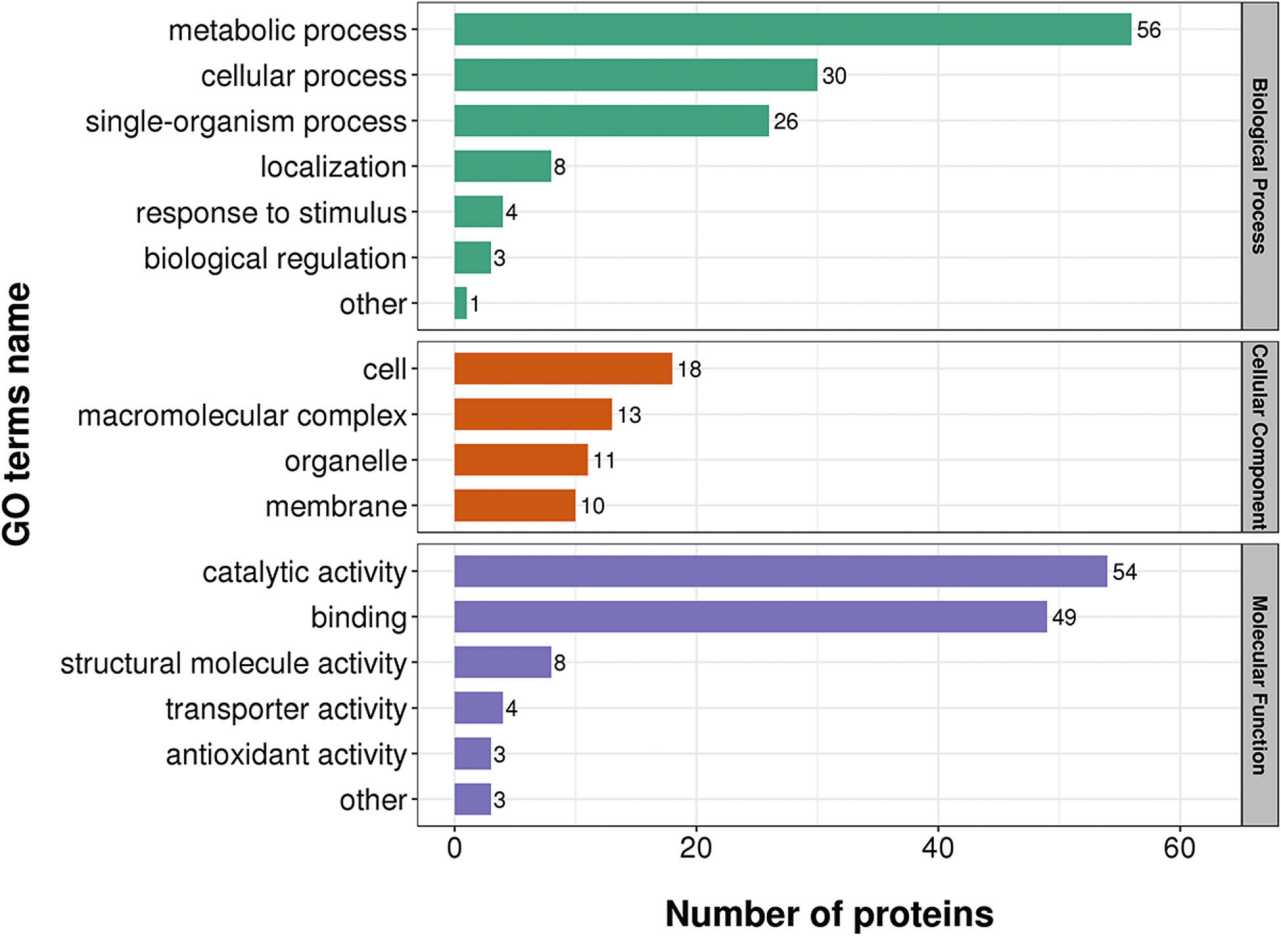
Gene Ontology (GO) classifications of differentially abundant proteins (DAPs) between the sterile line and its reproductive line. The x-axis represents the number of DAP proteins in each category, and the y-axis represents each GO term

Simultaneously, the cytoplasmic male Sterility also occurs mainly in mitochondria, and mitochondria are also used as a place for energy conversion in plant development. Therefore, we speculate that the occurrence of *pol* CMS may be related to the production and conversion of energy, thereby regulating the degradation of tapetum and triggering the development of anthers abnormal.” We performed GO analysis to determine the functional group of DAPs. The 140 DAPs between the CMS and the maintenance lines were divided into 17 functional groups, of which biological processes, cellular components, and molecular functions accounted for 7, 4, and 6 GO terms, respectively. For biological processes, there are 30 proteins in protein-rich cellular processes, 56 proteins in metabolic processes, 26 proteins related to a single biological process, four responses to stimulus, eight proteins for localization, and three proteins for regulation of the biological process. Besides, genes were restored in cytoplasmic male sterility in transport activation and further aspects of the existing changes. Related proteins involved in the cell cycle and restorer processes showed different *pol* CMS lines (Table S5). The cell components are disturbed; there are 18 cell component proteins, 11 organelles, 13 biological macromolecular complexes, and 10 Membrane-associated proteins. In the molecular functional group, proteins with catalytic and binding activities accounted for 54 and 49 proteins, respectively, four proteins had transporter active, eight structural molecular active proteins, three antioxidant proteins, and three other characteristic proteins (Fig. 5).

### Single-cell transcriptome sequencing of the tapetum of sterile and fertile lines of *pol* CMS near-isogenic lines

We used laser microdissection and Illumina technology for single-cell RNA-Seq analysis to clarify the relationship between differentially expressed genes (DEGs) and pol CMS in NILs. After raw data were trimmed, a total of 52,936,673 and 52,606,810 clean reads were obtained for fertile and sterile samples, respectively. Furthermore, the Q20 and Q30 were > 96.61 and > 92.53%, respectively. The GC content for both sterile and fertile samples was regularly shown to be approximately 45%, signifying that the sequencing was exact. All clean reads (105,543,483) were aligned using the Trinity program, resulting in 117,332 contigs with a mean length of 901 nt (Fig. S3). We performed clustering and identified 80,851 unigenes (> 200 bp); the average length was 1054 nt, and the N50 was 1586 nt. The length of all unigenes recorded was longer than 199 bp, and 86.95% of them ranged from 200 to 1999 bp. We subjected all assembled unigenes to search against the Nr, Swiss-Prot, and COG databases, and 66,143 (81.81%), 54,857 (67.85%), and 28,129 (34.79%) unigenes were aligned against these three protein databases, respectively (Fig. S3). Among them, 114,517 and 104,780 genes were detected in the sterile and restorer line materials, respectively, and 98,735 genes were detected in both materials.

**Fig. 6:**
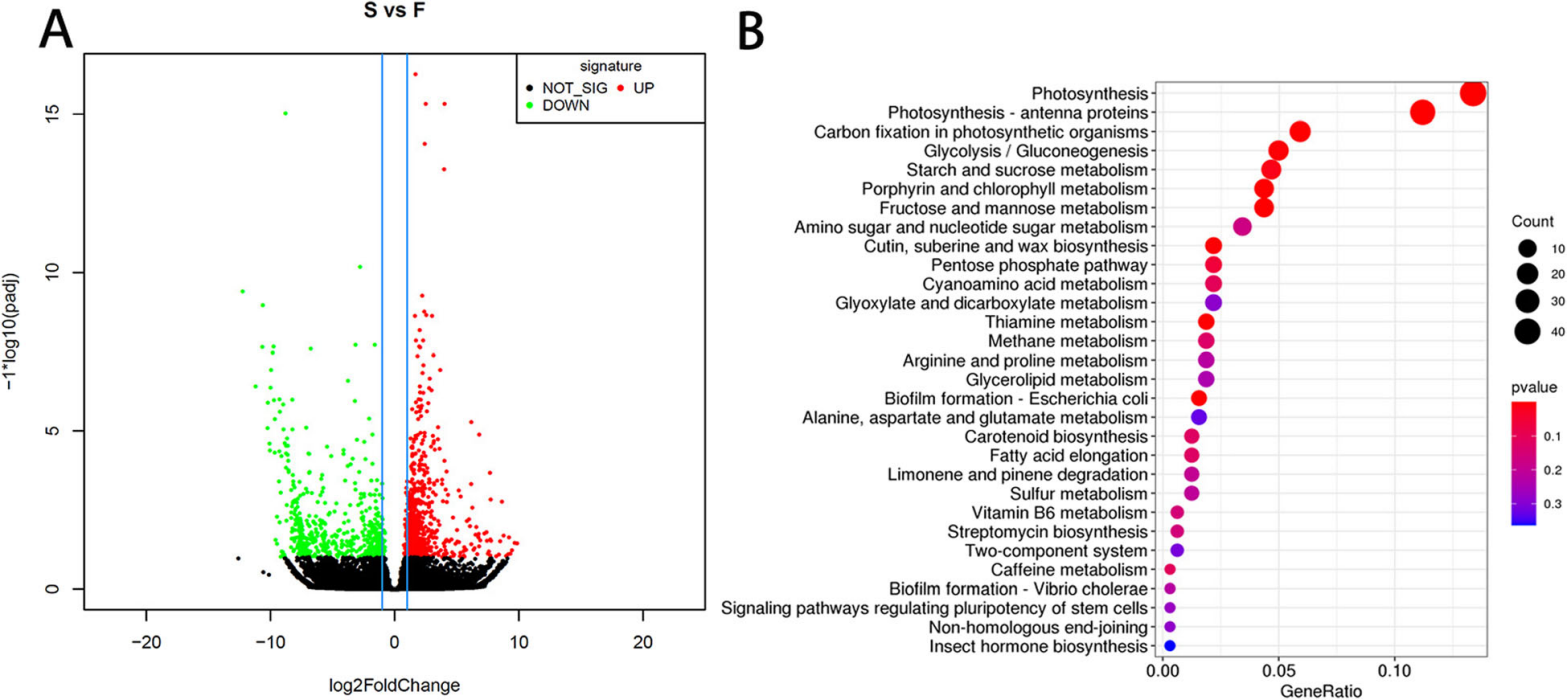
Transcriptome analysis results. **a** Volcano plot of the results of transcriptome analysis of significantly differentially expressed gene clusters. **b** KEGG pathway functional annotation gene distribution map of significantly differentially expressed genes

We analyzed DEGs between sterile and restorer lines for advanced recognition of differences in gene expression patterns. We identified 831 genes in the comparison of fertility and infertility, including 501 upregulated genes and 330 downregulated genes (Fig. 6a). The genes with significant differences of FDR ≤0.05 were selected for KEGG enrichment pathway analysis. The results showed enriched genes in Photosynthesis, Photosynthesis-antenna proteins, Carbon fixation in photosynthetic organisms, Glycolysis/Gluconeogenesis, Starch and sucrose metabolism, Porphyrin and chlorophyll metabolism, Fructose and mannose metabolism, Glycerophospholipid metabolism, and other metabolic pathways (Fig. 6b; Table S2, S6). Anther cells also showed rapid proliferation and vigorous metabolic processes during the growth process [37]. Therefore, the photosynthesis and sugar metabolism processes involved in energy metabolism may play an essential role in developing *B. napus* anthers.

### Combined transcriptome, proteome, and metabolome analysis

Through the combined analysis of proteomes, metabolomes, and transcriptomes, we found that in the sterile line and restorer line of *pol* CMS near-isogenic lines, owing to differences in the restoration gene *Rfp*, a series of genes and pathways occurred in *Brassica rapa*. We performed KEGG enrichment analysis on the detected DEGs and metabolites. The joint analysis of proteomes and metabolomes revealed a significant difference in glycolysis/gluconeogenesis and other metabolic pathways. The joint analysis of transcriptomes and metabolomes disclosed significant differences in three pathways: the phenylpropane biosynthesis pathway, glycolysis/gluconeogenesis metabolic pathway, and pyruvate biosynthesis pathway. However, transcriptome and metabolome enrichment analysis presented substantial differences in calcium-transporting ATPase activity, epithiospecifier protein, glycolysis/gluconeogenesis, phosphatase activity, and starch sucrose metabolisms (Fig. S4).

**Fig. 7:**
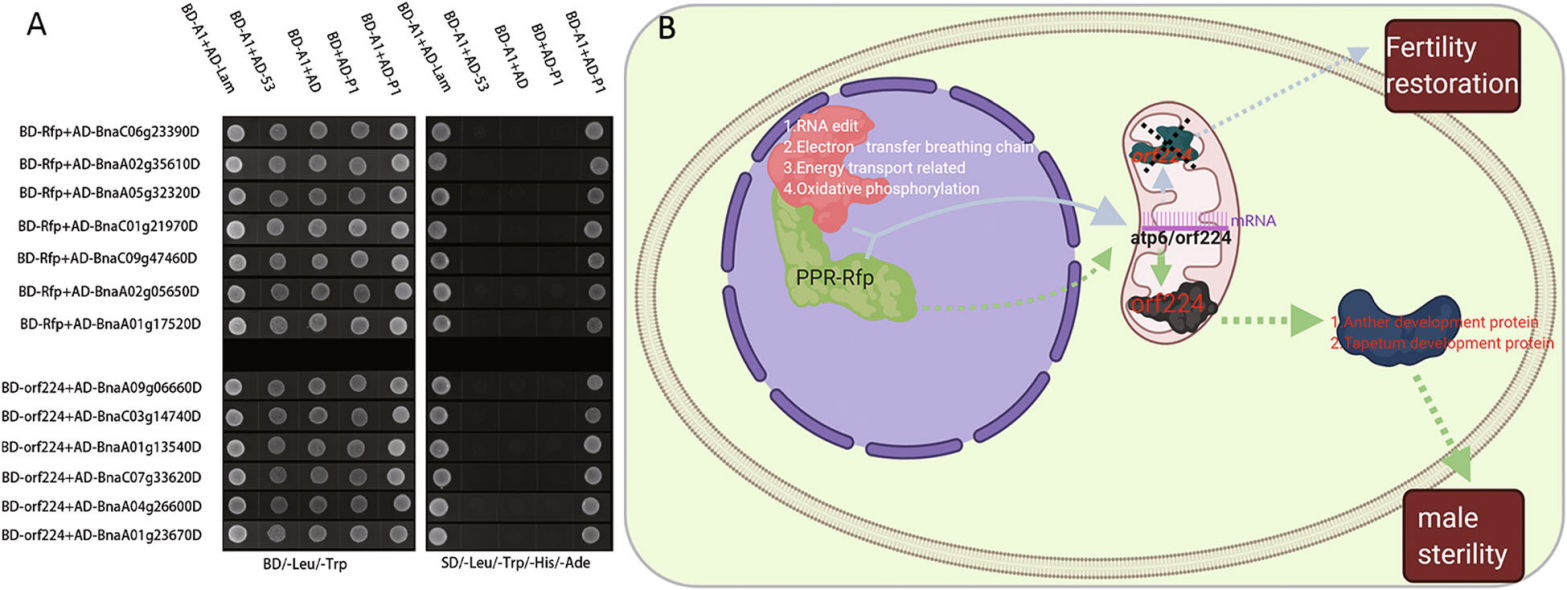
A Possible Mechanism Contributing to the Occurrence of Sterility and Recovery of Fertility in Pol CMS Lines of *B. napus*. **a** Multi-omics association analysis of candidate genes using yeast to screen for related genes. **b** A proposed a *pol* CMS working model showing the *pol* CMS mechanism and infertility mechanism of *Rfp* and *orf224*

In proteome, metabolome, and transcriptomics analysis, we obtained 141 differential proteins, 37 differential metabolites, and 831 differential genes. By combining the differential genes in the three omics of the proteome, transcriptome, and metabolome in the analysis, a total of 24 genes were screened for associated differences in the proteome, transcriptome, and metabolome. The functions of these genes mainly include genes related to RNA editing, respiratory electron transport chain, anther development protein, energy transport-related protein, tapetum development protein, and oxidative phosphorylation protein (Table 1). In the high-generation near-isogenic line materials, the significant differences in restoration genes led to differences in protein, metabolic, and transcription levels. Therefore, we screened for protein interactions between the *pol* CMS sterility gene *orf224* and the restoration gene *Rfp* by yeast two-hybridization to understand further the mechanisms of sterility and restoration genes (Fig. 7a). RT-PCR validated the genes validated by yeast two-hybridization, and only three genes were found to be insignificantly different, while all other genes were significantly different (Fig. S5).

**Table 1:** Screening of differentially expressed genes through joint analysis of multiple omics, followed by *Rfp* and *orf224* for yeast experiment verification

Transcription ID	Protein. accession	Protein interaction		log2FC	*p*-value	Description
		*orf*224	*Rfp*			
RNA editing protein						
BnaA09g06660D	A0A078FUQ6	+	–	−0.61706	0.00150	RNA recognition motif (RRM)-containing protein
BnaC06g23390D	A0A078FDM2	–	+	−0.23610	0.53657	RNA polymerase II subunit C-terminal domain phosphatase
BnaA01g22260D	A0A078GHA1	–	–	− 1.22769	0.01624	RNA-binding protein 25 isoforms X1
Respiratory electron transport chain protein						
BnaA02g35610D	A0A078JFK2	–	+	−0.79086	0.00062	Electron transfer activity/cupredoxin superfamily protein
BnaA05g32320D	A0A078I0S7	–	+	0.73948	0.16802	NADH dehydrogenase [ubiquinone] iron-sulfur protein 6
BnaA06g16370D	A0A078F502	–	–	0.98176	−0.00616	Gamma carbonic anhydrase-like 2
BnaC03g14740D	A0A078I202	+	–	−1.16106	0.00038	SPFH/Band 7/PHB domain-containing membrane protein
BnaC08g43300D	A0A078GBF1	–	–	−0.87303	0.00190	Encodes UDP-d-apiose synthase
BnaC09g47460D	A0A078G848	–	+	−1.00000	0.00098	NADH dehydrogenase [ubiquinone] flavoprotein 1
Anther development protein						
BnaA01g13540D	A0A078ICM7	+	–	−1.88014	0.00328	UDP-glucose 4-epimerase 2
BnaA02g05650D	A0A078F7X9	–	+	0.62574	0.00412	Pollen Ole e 1 allergen and extensin family protein
BnaA04g21720D	A0A078FVE9	–	–	− 4.52980	0.00001	Type III polyketide synthase A-like
BnaA08g15000D	A0A078GE34	–	–	−6.67917	0.00010	Tetraketide alpha-pyrone reductase 1-like
BnaC07g33620D	A0A078F9K9	+	–	0.83443	0.06855	Pollen-specific leucine-rich repeat extension-like protein 1
BnaA04g26600D	A0A078HGU6	+	–	−1.34373	0.00096	LDL receptor wingless signaling/trafficking chaperone
Energy transport-related protein						
BnaA03g01500D	A0A078FEE1	–	–	−1.18473	0.00094	Lysosomal beta glucosidase-like
BnaA10g11120D	A0A078HLN1	–	–	−1.42526	0.00070	Calcium-transporting ATPase
BnaC01g21970D	A0A078H9K8	–	–	−0.64160	0.04709	Dihydrolipoyl dehydrogenase
BnaC06g00540D	A0A078IEJ9	–	–	2.38727	0.00002	Probable mitochondrial chaperone BCS1-B
BnaCnng66500D	A0A078JT79	–	–	0.88088	0.00126	ATP synthase subunit delta, chloroplastic-like
Tapetum development protein						
BnaA01g23670D	A0A078FYQ2	+	–	−3.60823	0.00012	May play a role in tapetum development.
BnaA09g10680D	A0A078HQA1	+	–	−5.68514	0.00001	4-Coumarate-CoA ligase-like 1
Oxidative phosphorylation protein						
BnaA01g17520D	A0A078H3Z4	–	+	−2.71786	0.00052	Peroxidase
BnaC01g19630D	A0A078GUT8	–	–	1.71357	0.00002	NADPH-protochlorophyllide oxidoreductase

Studies have shown that the pathways related to RNA editing, respiratory electron transfer chain, anther development, energy transport, tapetum development, and oxidative phosphorylation involve interactions between infertility genes and restoration genes. We used yeast two-hybrid to find the protein that interacts with the restorer gene to analyze the mechanism by which the restorer genes restore fertility. The results showed that the restorer gene and the protein involved in RNA editing might work together in the *pol* CMS *atp6/orf224* transcript. Mutual work of the restorer gene and the protein involved in RNA editing was shown to induce the infertility gene to be non-functional. We also concluded that restorer genes also interacted with proteins related to energy, respiratory electron transfer chain, and pyruvate dehydrogenase, indicating that the occurrence of CMS and fertility restoration might be strictly associated with energy metabolism.

Similarly, we used the *orf224* protein of the sterility gene to verify the selected candidate genes. Previous studies have found that the sterility gene mainly interacted with pollen development and tapetum-related proteins; thus, we inferred that the sterility gene was mainly related to pollen development. The protein pathway related to tapetum development led to the occurrence of *pol* CMS. Therefore, considering our results above, we propose a hypothesis to explain the mechanism of *pol* CMS’s occurrence and the role of gene restoration. When the restorer gene is a stealth gene, it will not interfere with the sterile gene’s function. Therefore, the *orf224* protein would affect the proteins associated with the development of velvet cells or anther development in *B. napus,* thus resulting in anther abortion in *pol* CMS. When the restorer gene is dominant, as the restorer gene *Rfp* cannot directly cut the mRNA transcript, the *Rfp* gene will bind to the corresponding protein, which together causes the *atp6/orf226* transcript to change; this causes *orf224* to lose its toxic function, thereby restoring fertility.

## Discussion

With the advancement of science and technology, multi-omics analysis is extensively used to solve biological problems [38]. Multi-omics joint analysis can understand and prioritize the problems we need to solve from big data and multi-dimensional data [39]. This study used laser capture microdissection to extract RNA from tapetum cells for transcriptome analysis, and < 1 mm flower buds were used for proteomics and metabolomics analysis [29]. Excellent results were achieved for our subsequent analysis and screening of candidate genes. We obtained 24 candidate genes involved in RNA editing, electron transfer respiratory chain [16], anther development, energy conversion, tapetum development, and oxidative phosphorylation-associated through joint analysis of multi-omics pathways. Our results suggested that glycolysis/gluconeogenesis pathways were near related to the generation and transport of energy in the tricarboxylic acid cycle [40]. This study’s joint analysis found a correlation analysis of glycolysis/gluconeogenesis pathways is considerably diverse. Our findings refer that energy changes should be fundamental in *pol* CMS. Using the yeast two-hybrid system, we discovered the restorer gene, and it was found that the protein involved in RNA editing may work together in the *pol* CMS *atp6/orf224* transcript [29]. Furthermore, sharing caused the infertility gene to be non-functional.

The *pol* CMS restorer gene *Rfp* encodes a PPR protein that usually recognizes and binds to RNA sequences in organelles [39]. In a previous study of *pol* CMS, the materials of sterile, maintainer, and restorer lines were compared with northern blotting, and the results showed that the original *atp6/orf224* was transcribed into 1.1 kb, 2.2 kb, and 1.9 kb transcripts relative to the sterile and restorer lines, respectively. However, in the reintroduction line materials, two transcripts of 2.2 kb and 1.9 kb were significantly reduced, whereas two new transcripts of 1.4 kb and 1.3 kb appeared simultaneously [41]. Therefore, we presumed that *pol* CMS’s restoration gene participated in the editing of *atp6/orf224* mRNA. The transcript of *atp6/orf224* translates the *orf224* protein, and *orf224* has been found to exert a toxic effect on *Escherichia coli.*

Moreover*, orf288* has a toxic protein function in hau CMS, which leads to male sterility [2]. In the present study, we located three RNA editing-related proteins. Verification by yeast revealed that two proteins that interacted with restorer genes and one protein that interacted with sterile genes might be involved in cutting the *pol* CMS sterile gene *atp6/orf224* transcript (Fig. 7b).

Studies have found that CMS genes are mainly composed of mitochondrial genes, and mitochondria serve as the primary energy supplier in living organisms [[42, 43]]. The vast majority of CMS genes are chimeric genes generated by mitochondrial arrangement events, in which *cox1, atp8*, and *atp6* function as infertility genes; however, most of them encode electron transport respiratory chain-related proteins or ATP synthase related complexes [14, 44]. Co-transcription and the electron transport chain’s normal metabolism is closely related to energy metabolism [45]. It is believed that in maize T-CMS, T-URF13 causes unrecoverable damage near the MT membrane, leading to the loss of membrane potential and, ultimately, mitochondrial breakdown and sterility [[10, 46]]. In the rapeseed *ogu* CMS, the infertility gene *orf138* was found to disrupt the electron transport chain inside the mitochondrial membrane, leading to anthers abortive [47].

In the phenomenon of cytoplasmic male sterility in higher plants, mitochondrial gene mutations lead to infertility. Several crop studies have found that both early and delayed degradation of tapetum cells can lead to infertility. As a staged tissue, the tapetum provides nutrients and energy for the maturation of microspore cells. In the omics studies of Chinese cabbage [47], salvia [48], rape [49], watermelon [50], and cotton [51], it has been found that the glycolysis/gluconeogenesis pathway has an essential function in the process of male sterility. Because the glycolysis/gluconeogenesis pathway produces many carbohydrates and various enzymes, it is closely related to plants’ average growth and development. However, due to male sterility’s complex regulatory factors, this type of gene has not been verified by molecular biology for its specific effect on male sterility. However, through additional crop omics testing and analysis, the glycolysis/gluconeogenesis pathway is closely related to male sterility.

Similarly, the electron transport respiratory chain disorder and disrupted ATP synthase complex function have been found in the Petunia CMS Connett, wild G-CMS beets [42], and sunflower CMS [52]. These previous findings showed an association between the CMS protein and the mitochondrial electron transport chain complex. For example, in rice CMS lines, the sterility genes *orf79* and *orfH79* caused decreased ATP/ADP ratio and increased reactive oxygen species in anthers [15]. We found that the rapid division of mitochondrial genes during anther development may lead to mitochondrial function defects, leading to insufficient energy supply for male organ development and triggering abortion [52]. All the above information indicates that extreme energy insufficiency in mitochondria is the only possible cause of CMS, but the mechanism that triggers unfunctional pollen is still not entirely understood [53]. Researchers cogitated that the CMS protein binds to specific mitochondrial proteins in some way to achieve this effect, but no relevant candidate proteins have been found. Among our differential candidate genes, we also found electron transfer chain-related proteins and energy transfer-related proteins that interact with restorer genes, which may cause CMS and restorer of fertility [54]; however, further research and more scientific evidence are needed to confirm the current hypothesis.

We used stereomicroscopy and scanning electron microscopy to observe the differences in anther and pollen development in sterile and fertile lines of *B. napus*. The results showed that the pollen grains of sterile plants markedly shrank, bent inward, and collapsed, whereas those of fertile plants showed a normal phenotype [55]. In our research, using transmission electron microscopy analysis of semi-thin sections, we observed that *pol* CMS’s sterile line material exhibited abnormalities and disintegration. These abnormalities mostly occurred in the third phase of anther development and led to early PCD of sporogenous cells, which eventually led to the abnormal differentiation of L2 cells, outer cells, inner cells, tapetum cells, and microspore mother cells [54, 56, 57]. In this study, we identified many anther development-related proteins and tapetum development-related proteins in sterile plants, and these proteins were associated with sterility genes. Thus, we concluded that *pol* CMS’s sterility genes caused the production of abnormal anther and tapetum development-related proteins, which led to anther abortion [31]. However, there are also significant shortcomings in this project. Although several candidate genes were screened by combined multi-omics analysis and yeast two-hybrid experiments, the corresponding results were not verified by other molecular biology experiments and oil-seed rape transgenesis.

## Conclusions

In the combined analysis of *pol* CMS’s near-isogenic lines with multiple omics, we screened 24 differential genes and carried out yeast two-hybrid detection of these differential genes with the restorer gene *Rfp* sterility gene *orf224* in *pol* CMS, looking for the same *pol* CMS fertility changes related protein. Finally, we screened seven *Rfp* interacting proteins, whose primary functions are RNA editing, anther development, and tapetum development-related protein interactions. The sterility gene *orf224* protein screened out five interacting proteins; their main functions are electronic the respiratory transmission chain, anther development, and oxidative phosphorylation related proteins. Therefore, we speculate that the restorer gene *Rfp* acts on the sterility gene by forming a protein complex with the above types of proteins, resulting in the loss of function of the sterility gene and the restoration of fertility. Infertility genes mainly interact with proteins related to the electron respiratory transport chain, directly act on proteins related to anther development, or interact with proteins related to oxidative phosphorylation, which affect the development of anthers and fail to develop pollen grains that are initially normal. Therefore, we have screened 12 candidate genes through multi-omics joint analysis combined infertility genes and restorer genes for follow-up research, which provides a specific data basis for the analysis of *pol* CMS, and also provides help for revealing the potential molecular mechanism of *pol* CMS.

## Methods

### Sample collection

In the laboratory, bing409 (pol restorer line)(Source: Prof. Tingdong Fu) and 1141A (sterile pol line)(Source: Prof. Tingdong Fu) was used to establish a near-isogenic line (NIL) through 13 generations of backcrossing (Source: Dr. Zonghui Yang). Using this material, we finely mapped the *pol* CMS restorer gene. In the BC8 generation, the 32 kb infiltrated the specific marker scanning located gene fragment, and then the experiment was carried out by crossing 13 generations of sterile lines and reproductive lines. The NIL of oil-seed rape was seeded in May 2018 in Wuhan, with the seedling density controlled to 20 cm × 40 cm. The NIL material passed the multi-year backcrossing experiment, and the phenotype was 1:1 sterile line to the restorer line. The sterile and fertile phenotypes can be well distinguished. Through paraffin section observation, we found that the infertility period of *pol* CMS was 3–4 stages of anther development, and the degradation of tapetum occurred in advance, leading to abortion [29]. In stages 2–3 of anther development, the flower buds’ size was < 1 mm; therefore, we collected flower buds of < 1 mm and studied the proteome and metabolome. All samples taken were frozen in liquid nitrogen and stored at − 80 °C. Simultaneously, we collected the flower buds of sterile and fertile lines in the same period and fixed them for laser microdissection capture to study single-cell transcriptomics. This procedure was conducted to investigate *pol* CMS more accurately at the metabolic, protein, and transcription levels. The transcriptome, proteome, and metabolome sample collection set up three biological replicates for omics sequencing analysis.

### Metabolite extraction and processing

The sample preparation and metabolite extraction procedures were as follows: 100 ± 1 mg of 3–4-stage anther was put in a 2 mL EP tube, and 0.6 mL extraction buffer (V_methanol_: V_dH2O_ = 3:1) was added, followed by 20 μL of adonitol (1 mg/mL stock in dH_2_O) as per the internal standard. The column used was Waters ACQUITY UPLC HSS T3 C18 (1.8 μm, 2.1 mm × 100 mm. The mobile phase used was as follows: Phase A, ultrapure water (containing 0.04% acetic acid); Phase B, acetonitrile (containing 0.04% acetic acid). The elution gradient program was as follows: 0.00–10.00 min 0% B; 10.00–11.00 min 95% B; 11.00–11.10 min, 5% B; 11.10–14 min, maintained at 5% B.” At 10.00 min, B’s ratio increased linearly to 95% and remained at 95% for 1 min. From 11.00 to 11.10 min, B’s ratio decreased to 5% and maintained till 14 min. The elution conditions were as follows: The flow rate is 0.35 mL/min; the column temperature is 40 °C; the injection volume is 4 μL. The mass spectrometry conditions are as follows: the electrospray ionization temperature is 550 °C; the electrospray ionization temperature is 550 °C. Mass spectrometer voltage, 5500 V; curtain gas 30 psi; collision induced dissociation parameters [58]. Each ion pair is scanned based on the best intersecting potential and collision energy in the triple quadrupole for detection. Metabolic profiling is performed using a broad range of targeted metabolomic approaches at Wuhan Metware Biotechnology [59]. (Wuhan, China) (http://www.metware.cn/).

### Lab-free method for proteome detection

The anther was first ground in liquid nitrogen. The powder was transferred to a 5 mL centrifuge tube and sonicated three times on ice using a high-intensity ultrasonic processor (Sanchez-Puerto et al., 2015) in lysis buffer (containing 1% TritonX-100, 10 mM dithiothreitol, 1% protease inhibitor cocktail, 50 μM PR-619, 3 μM TSA, 50 mM NAM, and 2 mM EDTA). Equal volume of trisaturated phenol (ph 8.0) was added, and then the mixture was further rotated for 5 min. After centrifugation (4 °C, 10 min, 5000×g), the upper phenol phase was transferred to a new centrifuge tube. Proteins were precipitated by adding at least four volumes of ammonium sulfate-saturated methanol, followed by incubation at − 20 °C for at least 6 h. After centrifugation at 4 °C for 10 min, the supernatant was discarded. The remaining precipitate was washed once with ice-cold methanol and then with ice-cold acetone three times. Re-dissolve the protein in 8 M urea and use the BCA kit to determine the protein concentration according to the manufacturer’s instructions.

The trypsin peptide was dissolved in 0.1% formic acid (solvent a) and directly loaded on a self-made reversed-phase analytical column (length: 15 cm, 75 μ m). The gradient consisted of increasing from 6% B to 23% solvent B (0.1% formic acid in 98% acetonitrile) in 26 min, from 23 to 35% in 8 min, climbing to 80% in 3 min, and then remaining at 80% on easy-nlc 1000 UPLC system. The flow rate was 400 nl / min in the last 3 min. The peptides were processed from NSI sources, and then tandem mass spectrometry (MS/MS) was performed in Q ExactiveTM Plus (Thermo) connected online to UPLC. The applied electrospray voltage was 2.0 kV. The m/z scan range of the complete scan is 350 to 1800, and the complete peptide is detected in Orbitrap with a resolution of 70,000. Then use NCE to set 28 to select peptides for MS/MS, and detect these fragments in Orbitrap with a resolution of 17,500. The data-dependent process performs 20 MS/MS scans after one MS scan, and the dynamic elimination time is 15.0 s. Automatic gain control (AGC) is set to 5E4. The fixed first mass setting is 100 m/z [60].

The corresponding signal abundance of the protein in each sample is detected by mass spectrometry technology. The LFQ intensity of the protein in each sample is obtained by the non-standard quantitative calculation method, and the relative quantitative value of each sample is obtained according to the protein LFQ intensity between different samples. The first step is to calculate the protein’s differential expression between the two samples in the comparison group. First, calculate the average of each sample’s quantitative value in multiple replicates, and then calculate the ratio of the average between the two samples, which is taken as the final differential expression of the comparison group. The second step is to calculate the significance *P*-value of the protein’s differential expression in the two samples. First, take the relative quantitative value of each sample to log2 (to make the data conform to the normal distribution), and then use the two-sample two-tailed T-test method to calculate a *p*-value. When *p*-value< 0.05, the change of differential expression exceeding 1.5 is used as the change threshold for significant up-regulation, and the change threshold for significant down-regulation is less than 1/1.5.

Gene was classified by Gene Ontology annotation into three categories: biological process, cellular compartment, and molecular function. The GO with a corrected *p* value of < 0.05 is considered important [61]. The Encyclopedia of Genes and Genomes (KEGG) database is used to identify enrichment pathways through two-tailed Fisher’s exact test to test the enrichment of all identified proteins by differentially expressed proteins [62]. Paths with corrected *p*-value < 0.05 are considered important.

### Single-cell transcriptome sequencing using microdissection

Laser microdissection technology was used to separate single cells or organelles from tissue sections by laser cutting. The operator observed a tissue slice under a microscope, located the target cell, marked the target cell area on the computer display screen, and then cut the region with the laser to extract it [52].

First, the flower bud sections were treated with 75, 85, and 95% alcohol in a water bath of 58 °C each for 90 s each, followed by n-butanol and absolute ethanol at varying ratios 1:1 and 3:1 at 58 °C twice for 90 s each. Finally, the pre-embedding treatment of the micro-cut material was completed by treatment with n-butanol at 58 °C for 90 s. Subsequently, the sample was immersed in paraffin at 65 °C three times for 30 min, 60 min, and 90 min each, and then embedded in an embedding box. Slides with glass slide membrane were treated with RNase and 75% alcohol. The thickness of the sections was selected according to the depth of the treated material. The flower bud width was sliced 16 μm.

The single-cell samples were collected in tubes containing lysis components and ribonuclease inhibitors. Amplification was then performed by using the Smart-Seq2 method [63]. An Oligo-dT primer was introduced to the reverse transcription reaction for first-strand cDNA synthesis, followed by PCR amplification to enrich the cDNA and a magnetic bead purification step to clean up the synthesized material. Next, the cDNA product was checked by a Qubit® 3.0 Fluorometer and Agilent 2100 Bioanalyzer (Thermo Fisher Scientific) to ensure the expected output of length approximately 1–2 kbp. Subsequently, cDNA was sheared randomly by ultrasonic waves for Illumina library preparation, including DNA fragmentation, end-repair, 3′ end A-tailing, adapter ligation, PCR amplification, and library validation. PerkinElmer LabChip® GX Touch and Step OnePlus™ Real-Time PCR System were introduced for library quality inspection after library preparation. Qualified libraries were then loaded on the Illumina Hiseq platform for PE150 sequencing.

### Yeast two-hybrid assay

The full-length cDNAs of the recovery gene *Rfp* and the sterility gene *orf224* were amplified and cloned into the bait vector pGBKT7, while the candidate genes were cloned into the prey vector pGADT7, respectively. The vectors constructed by bait vector pGBKT7 and prey vector pGADT7 were then co-transformed into yeast strain AH109 receptor cells [64].

### RNA extraction, reverse transcription PCR

Referring to the reagent manufacturer’s instructions, we extracted total RNA from kale type oilseed rape using Trizol reagent (Invitrogen), resuspended the RNA in RNase-free water, and treated it with RNase-free DNase I (Thermo Fermentas). After digestion treatment with DNase I, PCR amplification was used to confirm the elimination of DNA contamination. We then reverse transcribed the digested RNA using M-MLV reverse transcriptase (Invitrogen) and random primers (Thermo Fermentas) to obtain cDNA. cDNA was amplified on a Light Cycler 480 (Roche) using SYBR Green I with gene-specific primers. Real-time quantitative PCR was performed on a Light Cycler 480 (Roche) using a SYBR Green I Master PCR kit with gene-specific primers to detect the expression of kale type oilseed rape genes [65].

## Supplementary Information

**Additional file 1: Figure S1.** Metabolome heat map and KEGG enrichment analysis statistical map (A) Heat map of clustering of significant metabolites of sterile lines and restore lines of near-isogenic lines. (B) Analysis of KEGG enrichment of significant metabolites

**Additional file 2: Figure S2.** Proteome analysis of *pol* CMS near-isogenic lines and sterile lines. (A) Basic statistical graph of proteome mass spectrometry data results. (B) PCA analysis of sterile proteome line and restore line material. (C) Proteome mass spectrometry analysis of the overall peptides. (D) Differentially expressed proteins

**Additional file 3: Figure S3.** Quality summary of single-cell transcriptome original sequencing data

**Additional file 4: Figure S4.** (A) Single-cell transcriptome Venn diagram shows the expression of genes between samples. (B) Proteome and metabolome combined analysis with KEGG enrichment analysis. (C) Proteome and transcriptome joint analysis with KEGG enrichment analysis. (D) Combined analysis of transcriptome and metabolome with KEGG enrichment

**Additional file 5: Figure S5.** RT-PCR was used to detect candidate genes’ relative expression levels in pol CMS sterile 6330A and restorer lines

**Additional file 6: Table S1.** Significantly different metabolites of the metabolome

**Additional file 7: Table S2.** Significantly different genes of transcription

**Additional file 8: Table S3.** Significantly different proteins of proteome

**Additional file 9: Table S4.** Enrichment analysis of KEGG metabolites

**Additional file 10: Table S5.** Proteomic Differential Protein KEGG Enrichment Analysis

**Additional file 11: Table S6.** Transcriptome differential gene KEGG enrichment analysis

## Data Availability

The original transcriptome sequencing data related to this article is stored in the NCBI SRA database with the Submission ID SUB8446872 (https://submit.ncbi.nlm.nih.gov/subs/bioproject/) and the BioProject ID PRJNA673655(https://www.ncbi.nlm.nih.gov/sra/PRJNA673655), Reviewer link: https://dataview.ncbi.nlm.nih.gov/object/PRJNA673655?reviewer=5lfjtb644f2dg76db7tat7pvvh . Other data from the results of this article are also included in this article and its attached files.

## Abbreviations

PPR: Pentatricopeptide Repeat
CMS: Cytoplasmic male sterility
PCD: Programmed cell death
GO: Gene Ontology
KEGG: The Encyclopedia of Genes and Genomes
PCA: Principal component analysis
DAPs: Differentially abundant proteins
DEGs: Differentially expressed genes
ESP: Epithiospecifier protein

## References

[1] Eckardt NA (2006). Cytoplasmic male sterility and fertility restoration. Plant Cell.

[2] Heng S, Gao J, Wei C, Chen F, Li X, Wen J (2018). Transcript levels of *orf288* are associated with the hau cytoplasmic male sterility system and altered nuclear gene expression in Brassica juncea. J Exp Bot.

[3] Chase CD, Gabay-Laughnan S. Cytoplasmic Male Sterility and Fertility Restoration by Nuclear Genes BT- Molecular Biology and Biotechnology of Plant Organelles: Chloroplasts and mitochondria. 2004. p. 593–621.

[4] Chase CD (2007). Cytoplasmic male sterility: a window to the world of plant mitochondrial–nuclear interactions. Trends Genet.

[5] Hanson MR, Bentolila S. Interactions of mitochondrial and nuclear genes that affect male gametophyte development. Plant Cell. 2004;16 Suppl Suppl: S154–S169. 10.1105/tpc.015966 PMC2643387 15131248

[6] Mackenzie S. 12 - Male sterility and hybrid seed production. 2012. p. 185–194.

[7] Chen L, Liu Y-G. Male sterility and fertility restoration in crops. Annu Rev Plant Biol. 2013;65. 10.1146/annurev-arplant-050213-040119 24313845

[8] Wang Z, Zou Y, Li X, Zhang Q, Chen L, Wu H, et al. Cytoplasmic Male Sterility of Rice with *Boro II* Cytoplasm Is Caused by a Cytotoxic Peptide and Is Restored by Two Related PPR Motif Genes via Distinct Modes of mRNA Silencing. Plant Cell. 2006;18:676 LP – 687. 10.1105/tpc.105.038240 PMC1383642 16489123

[9] Jing B, Heng S, Tong D, Wan Z, Fu T, Tu J, et al. A male sterility-associated cytotoxic protein *ORF288* in *Brassica juncea* causes aborted pollen development. J Exp Bot. 2012:63. 10.1093/jxb/err355 PMC3276091 22090439

[10] Korth KL, Kaspi CI, Siedow JN, Levings CS (1991). URF13, a maize mitochondrial pore-forming protein, is oligomeric and has a mixed orientation in *Escherichia coli* plasma membranes. Proc Natl Acad Sci U S A.

[11] Luo D, Xu H, Liu Z, Guo J, Li H, Chen L, et al. A detrimental mitochondrial-nuclear interaction causes cytoplasmic male sterility in rice. Nat Genet. 2013;45. 10.1038/ng.2570 23502780

[12] Lee SJ, Warmke HE (1979). Organelle size and number in fertile and T-cytoplasmic male-sterile corn. Am J Bot.

[13] Logan DC (2006). The mitochondrial compartment. J Exp Bot.

[14] Rhoads DM, Levings CS, Siedow JN (1995). URF13, a ligand-gated, pore-forming receptor for T-toxin in the inner membrane of cms-T mitochondria. J Bioenerg Biomembr.

[15] Peng X, Wang K, Hu C, Zhu Y, Wang T, Yang J (2010). The mitochondrial gene *orfH79* plays a critical role in impairing both male gametophyte development and root growth in CMS-Honglian rice. BMC Plant Biol.

[16] Wang K, Gao F, Ji Y, Liu Y, Dan Z, Yang P (2013). *ORFH79* impairs mitochondrial function via interaction with a subunit of electron transport chain complex III in Honglian cytoplasmic male sterile rice. New Phytol.

[17] Ogura H. Studies on the new male-sterility in Japanese radish, with special reference to the utilization of this sterility towerds the practical raising of hybrid seeds. 1967.

[18] Thompson KF (1972). Cytoplasmic male-sterility in oil-seed rape. Heredity (Edinb).

[19] Banga SS, Labana KS, Banga SK (1984). Male sterility in Indian mustard *(Brassica juncea L.)* Coss a biochemical characterization. Theor Appl Genet.

[20] Hu Q, Hua W, Yin Y, Zhang X, Liu L, Shi J, et al. Rapeseed research and production in China. Crop J. 2016:5.

[21] Liu J, Li M, Wang H, Yu L, Li D (2010). Sequence analysis and expression of *orf224* gene associated with two types of cytoplasmic male sterility in *Brassica napus L*. Z Naturforsch C.

[22] Heng S, Wei C, Jing B, Wan Z, Wen J, Yi B (2014). Comparative analysis of mitochondrial genomes between the hau cytoplasmic male sterility (CMS) line and its iso-nuclear maintainer line in *Brassica juncea* to reveal the origin of the CMS-associated gene *orf288*. BMC Genomics.

[23] Liu J, Xiang R, Wang W, Mei D, Li Y, Mason A, et al. Cytological and molecular analysis of Nsa CMS in *Brassica napus L*. Euphytica. 2015:206.

[24] Hu Q, Andersen S, Dixelius C, Hansen L (2002). Production of fertile intergeneric somatic hybrids between *Brassica napus* and Sinapis arvensis for the enrichment of the rapeseed gene pool. Plant Cell Rep.

[25] Li P, Kang L, Wang A, Cui C, Jiang L, Guo S, et al. Development of a fertility restorer for inap CMS (Isatis indigotica) *Brassica napus* through genetic introgression of one alien addition. Front Plant Sci. 2019:10. 10.3389/fpls.2019.00257 PMC6412144 30891056

[26] L'Homme Y, Stahl RJ, Li X-Q, Hameed A, Brown GG (1997). *Brassica napus* cytoplasmic male sterility is associated with expression of a mtDNA region containing a chimeric gene similar to the pol CMS-associated *orf224* gene. Curr Genet.

[27] Wei W, Wang H, Liu G (2007). Transcriptional regulation of 10 mitochondrial genes in different tissues of NCa CMS system in *Brassica napus L*. and their relationship with sterility. J Genet Genomics.

[28] Handa H, Gualberto JM, Grienenberger J-M (1995). Characterization of the mitochondrial *orfB* gene and its derivative, *orf224*, a chimeric open reading frame specific to one mitochondrial genome of the "Polima" male-sterile cytoplasm in rapeseed *(Brassica napus L.)*. Curr Genet.

[29] Liu Z, Yang Z, Wang X, Li K, An H, Liu J, et al. A Mitochondria-Targeted PPR Protein Restores *pol* Cytoplasmic Male Sterility by Reducing *orf224* Transcript Levels in Oilseed Rape. Mol Plant. 9:1082–4. 10.1016/j.molp.2016.04.004 27102212

[30] Liu Z, Liu P, Long F, Hong D, He Q, Yang G (2012). Fine mapping and candidate gene analysis of the nuclear restorer gene *Rfp* for *pol* CMS in rapeseed *(Brassica napus L.)*. Theor Appl Genet.

[31] Liu Z, Dong F, Wang X, Wang T, Su R, Hong D (2017). A pentatricopeptide repeat protein restores *nap* cytoplasmic male sterility in *Brassica napus*. J Exp Bot.

[32] Sheoran I, Sawhney V (2010). Proteome analysis of the normal and Ogura (ogu) CMS anthers of *Brassica napus* to identify proteins associated with male sterility. Botany..

[33] An H, Yang Z, Yi B, Wen J, Shen J, Tu J (2014). Comparative transcript profiling of the fertile and sterile flower buds of *pol* CMS in *B napus*. BMC Genomics.

[34] Rabinowitz JS, Robitaille AM, Wang Y, Ray CA, Thummel R, Gu H (2017). Transcriptomic, proteomic, and metabolomic landscape of positional memory in the caudal fin of zebrafish. Proc Natl Acad Sci U S A.

[35] Ichihashi Y, Date Y, Shino A, Shimizu T, Shibata A, Kumaishi K, et al. Multi-omics analysis on an agroecosystem reveals the significant role of organic nitrogen to increase agricultural crop yield. Proc Natl Acad Sci. 2020;117:14552 LP – 14560. 10.1073/pnas.1917259117 PMC7321985 32513689

[36] Hwang B, Lee JH, Bang D (2018). Single-cell RNA sequencing technologies and bioinformatics pipelines. Exp Mol Med.

[37] Zhang D, Yang L (2014). Specification of tapetum and microsporocyte cells within the anther. Curr Opin Plant Biol.

[38] Satoh M, Kubo T, Nishizawa S, Estiati A, Itchoda N, Mikami T (2004). The cytoplasmic male-sterile type and normal type mitochondrial genomes of sugar beet share the same complement of genes of known function but differ in the content of expressed ORFs. Mol Gen Genomics.

[39] Tagliamonte MS, Waugh SG, Prosperi M, Mai V (2019). An integrated approach for efficient multi-Omics joint analysis ACM Conf bioinformatics, Comput biol biomed ACM Conf bioinformatics. Comput Biol Biomed.

[40] Guasch-Ferré M, Santos JL, Martínez-González MA, Clish CB, Razquin C, Wang D (2020). Glycolysis/gluconeogenesis and tricarboxylic acid cycle-related metabolites, Mediterranean diet, and type 2 diabetes. Am J Clin Nutr.

[41] L'Homme Y, Stahl RJ, Li XQ, Hameed A, Brown GG. *Brassica napus* cytoplasmic male sterility is associated with expression of a mtDNA region containing a chimeric gene similar to the *pol* CMS-associated *orf224* gene. Curr Gen. 1997:31. 10.1007/s002940050212 9108140

[42] Ducos E, Touzet P, Boutry M (2001). The male sterile G cytoplasm of wild beet displays modified mitochondrial respiratory complexes. Plant J.

[43] Bergman P, Edqvist J, Farbos I, Glimelius K (2000). Male-sterile tobacco displays abnormal mitochondrial *atp1* transcript accumulation and reduced floral ATP/ADP ratio. Plant Mol Biol.

[44] Arrieta-Montiel M, Mackenzie S. Plant Mitochondrial Genomes and Recombination. In: Advances in Plant Biology: Plant Mitochondria. 2011. p. 65–82.

[45] Kubo T, Newton KJ (2008). Angiosperm mitochondrial genomes and mutations. Mitochondrion..

[46] Grelon M, Budar F, Bonhomme S, Pelletier G (1994). Ogura cytoplasmic male-sterility (CMS)-associated *orf138* is translated into a mitochondrial membrane polypeptide in male-sterile Brassica cybrids. Mol Gen Genet MGG.

[47] Zhou X, Shi F, Zhou L (2019). iTRAQ-based proteomic analysis of fertile and sterile flower buds from a genetic male sterile line 'AB01'in Chinese cabbage *Brassica campestris* L. ssp. pekinensis. J Proteome.

[48] Wang R, Lu C, Shu Z (2020). iTRAQ-based proteomic analysis reveals several key metabolic pathways associated with male sterility in Salvia miltiorrhiza. RSC Adv.

[49] Zhiming S, Zhen W, Xiaoqian M (2012). A dominant gene for male sterility in Salvia miltiorrhiza Bunge [J]. PLoS One.

[50] Heng S, Chen F, Wei C (2019). Cytological and iTRAQ-based quantitative proteomic analyses of hau CMS in Brassica napus L. J Proteome.

[51] Wang Y, Yang X, Yadav V (2020). Analysis of differentially expressed genes and pathways associated with male sterility lines in watermelon via bulked segregant RNA-seq. 3 Biotech.

[52] Balk J, Leaver CJ (2001). The PET1-CMS mitochondrial mutation in sunflower is associated with premature programmed cell death and cytochrome c release. Plant Cell.

[53] de BIP, Troxell RM, Graves JS (2019). Mitochondrial Dysfunction and Multiple Sclerosis. Biology (Basel).

[54] Hu J, Wang K, Huang W, Liu G, Gao Y, Wang J (2012). The rice pentatricopeptide repeat protein RF5 restores fertility in Hong-Lian cytoplasmic male-sterile lines via a complex with the glycine-rich protein GRP162. Plant Cell.

[55] Yuan M, Yang G-S, Fu T-D, Li Y (2003). Transcriptional control of *orf224/atp6* by the *pol* CMS restorer *Rfp* gene in *Brassica napus L*. Yi Chuan Xue Bao.

[56] Streets AM, Zhang X, Cao C, Pang Y, Wu X, Xiong L (2014). Microfluidic single-cell whole-transcriptome sequencing. Proc Natl Acad Sci U S A.

[57] Stévant I, Nef S (2018). Single cell transcriptome sequencing: a new approach for the study of mammalian sex determination. Mol Cell Endocrinol.

[58] Wang L, Liang W, Xing J (2013). Dynamics of chloroplast proteome in salt-stressed mangrove Kandelia candel (L.) Druce. J Proteome Res.

[59] Li H, Wu L, Tang N (2020). Analysis of transcriptome and phytohormone profiles reveal novel insight into ginger (Zingiber officinale rose) in response to postharvest dehydration stress. Postharvest Biol Technol.

[60] Wang Z, Yang X, Liu C, Li X, Zhang B, Wang B, Zhang Y, Song C, Zhang T, Liu M (2019). Acetylation of PHF5A modulates stress responses and colorectal carcinogenesis through alternative splicing-mediated Upregulation of KDM3A. Mol Cell.

[61] Chen G, Ye X, Zhang S (2018). Comparative Transcriptome analysis between fertile and CMS flower buds in Wucai (Brassica campestris L.). BMC Genomics.

[62] Zhang Q, Xu Y, Huang J, et al. The Rice Pentatricopeptide repeat protein PPR756 is involved in pollen development by affecting multiple RNA editing in mitochondria. Front Plant Sci. 2020:11. 10.3389/fpls.2020.00749 PMC7303307 32595669

[63] Chen P, Liao J, Huang Z, Li R, Zhao Y, Ran S (2014). Comparative proteomics study on anther mitochondria between cytoplasmic male sterility line and its maintainer in Kenaf *(Hibiscus cannabinus L.)*. Crop Sci.

[64] Sui X, Hu Y, Ren C (2020). METTL3-mediated m 6 A is required for murine oocyte maturation and maternal-to-zygotic transition. Cell cycle (Georgetown, Tex.).

[65] Hu YM, Tang JH, Yang H, Xie HL, Lu XM, Niu JH (2006). Identification and mapping of *Rf-I* an inhibitor of the *Rf5* restorer gene for Cms-C in maize *(Zea mays L.)*. Theor Appl Genet.

