# Supplementary figures and images for: High-generation near-isogenic lines combined with multi-omics to study the mechanism of polima cytoplasmic male sterility
Source: BMC Plant Biol. 2021 Mar 5;21:130. doi: 10.1186/s12870-021-02852-7 (PMC7934456; doi:10.1186/s12870-021-02852-7)

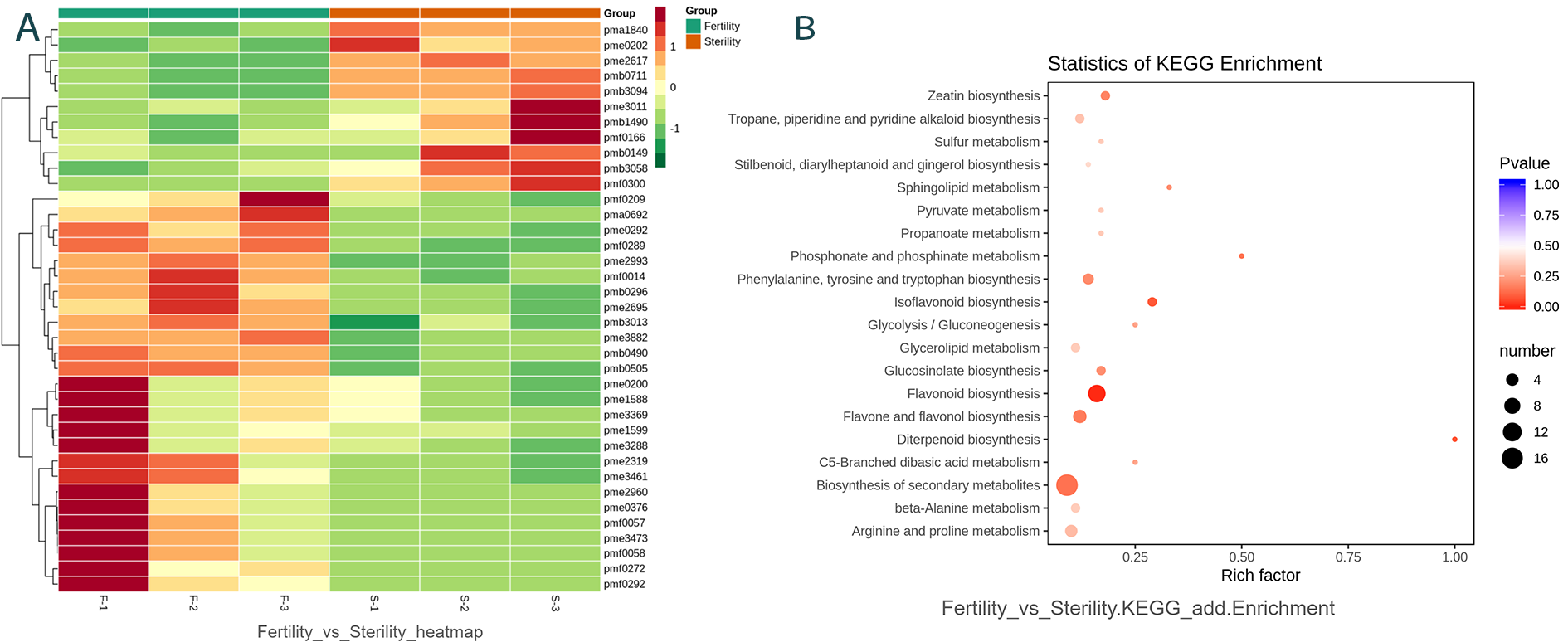

Supplement: Supplementary file 1 — Additional file 1: Figure S1. Metabolome heat map and KEGG enrichment analysis statistical map (A) Heat map of clustering of significant metabolites of sterile lines and restore lines of near-isogenic lines. (B) Analysis of KEGG enrichment of significant metabolites [file 12870_2021_2852_MOESM1_ESM.tif]

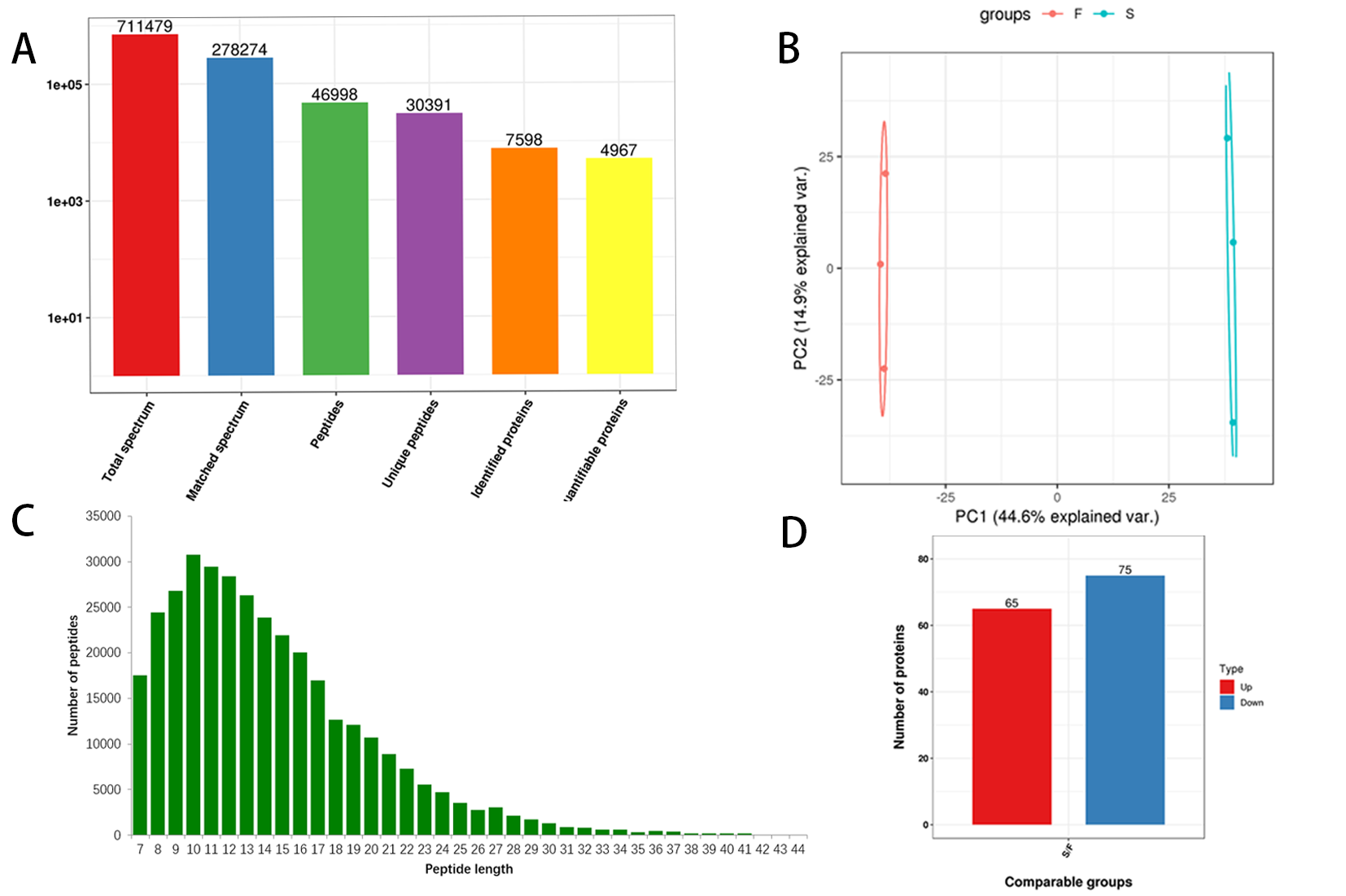

Supplement: Supplementary file 2 — Additional file 2: Figure S2. Proteome analysis of pol CMS near-isogenic lines and sterile lines. (A) Basic statistical graph of proteome mass spectrometry data results. (B) PCA analysis of sterile proteome line and restore line material. (C) Proteome mass spectrometry analysis of the overall peptides. (D) Differentially expressed proteins [file 12870_2021_2852_MOESM2_ESM.tif]

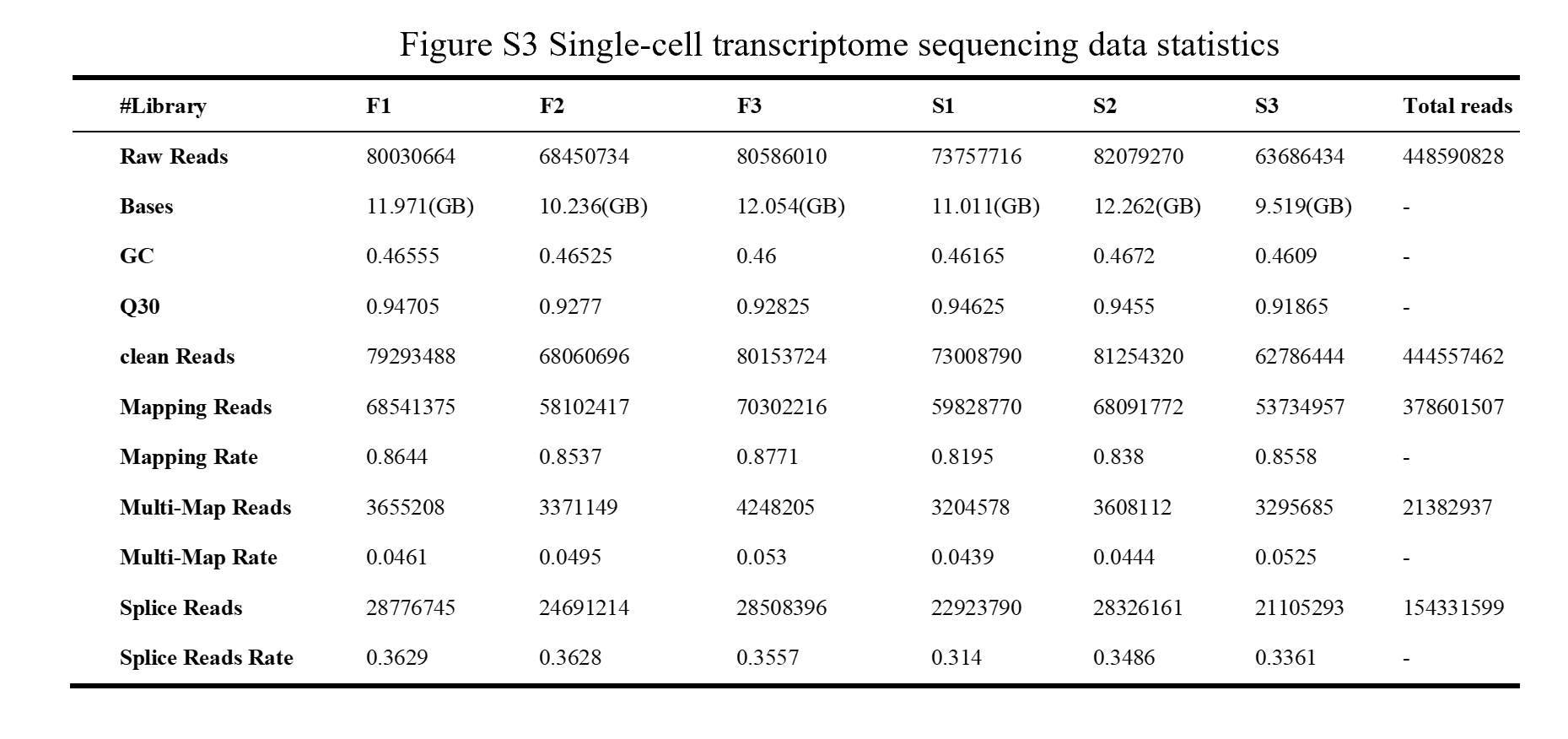

Supplement: Supplementary file 3 — Additional file 3: Figure S3. Quality summary of single-cell transcriptome original sequencing data [file 12870_2021_2852_MOESM3_ESM.png]

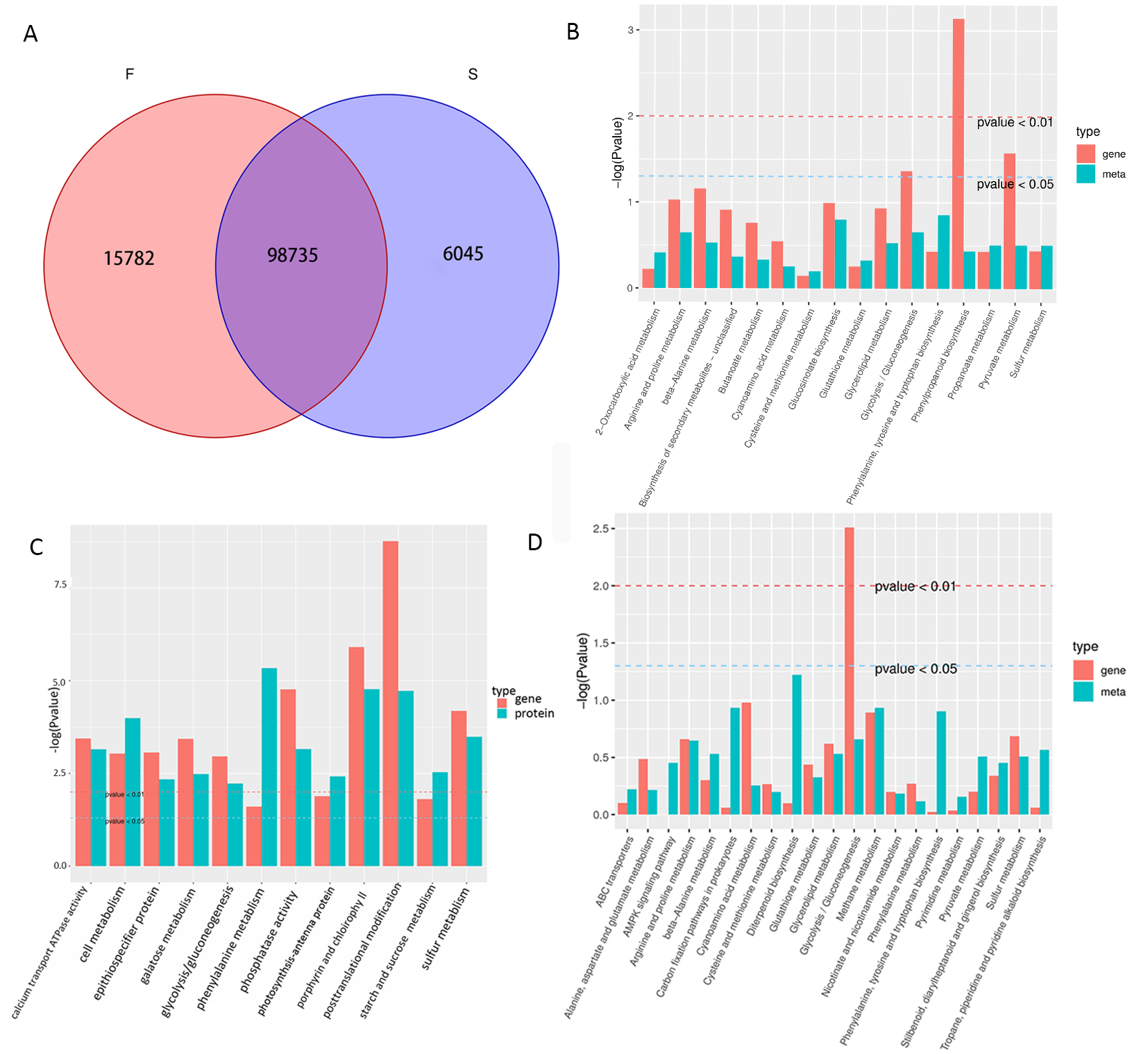

Supplement: Supplementary file 4 — Additional file 4: Figure S4. (A) Single-cell transcriptome Venn diagram shows the expression of genes between samples. (B) Proteome and metabolome combined analysis with KEGG enrichment analysis. (C) Proteome and transcriptome joint analysis with KEGG enrichment analysis. (D) Combined analysis of transcriptome and metabolome with KEGG enrichment [file 12870_2021_2852_MOESM4_ESM.tif]

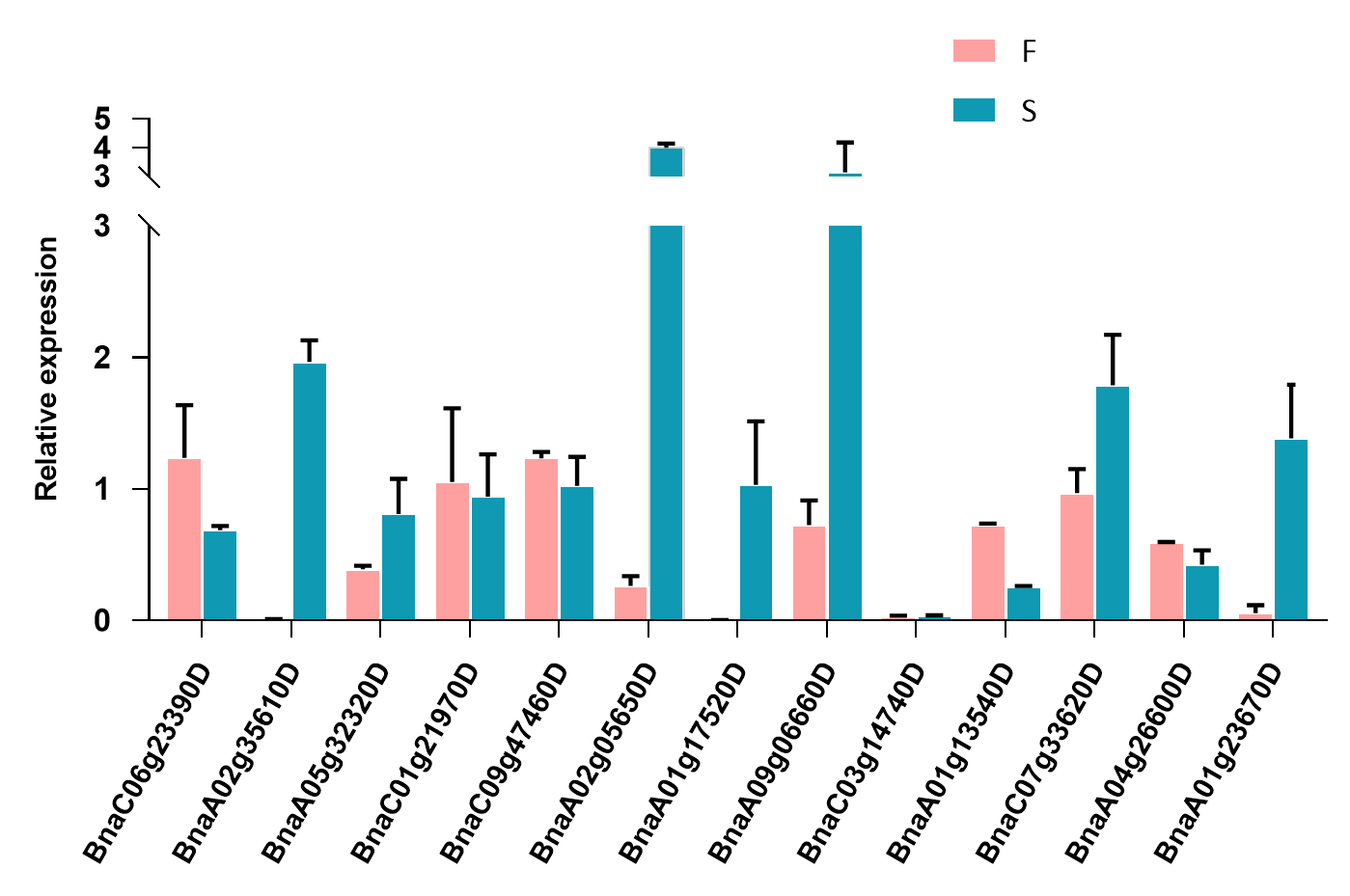

Supplement: Supplementary file 5 — Additional file 5: Figure S5. RT-PCR was used to detect candidate genes’ relative expression levels in pol CMS sterile 6330A and restorer lines [file 12870_2021_2852_MOESM5_ESM.png]
